# Probing the Response of the Amphibious Plant *Butomus umbellatus* to Nutrient Enrichment and Shading by Integrating Eco-Physiological With Metabolomic Analyses

**DOI:** 10.3389/fpls.2020.581787

**Published:** 2020-12-16

**Authors:** Paraskevi Manolaki, Georgia Tooulakou, Caroline Urup Byberg, Franziska Eller, Brian K. Sorrell, Maria I. Klapa, Tenna Riis

**Affiliations:** ^1^Aarhus Institute of Advanced Studies, AIAS, Aarhus, Denmark; ^2^Department of Biology-Aquatic Biology, Aarhus University, Aarhus, Denmark; ^3^Metabolic Engineering and Systems Biology Laboratory, Institute of Chemical Engineering Sciences, Foundation for Research & Technology-Hellas (FORTH/ICE-HT), Patras, Greece

**Keywords:** ecophysiology, nutrient enrichment, abiotic plant stresses, systems biology, shading effect, eco-metabolomics, Butomus umbellatus, amphibious plants

## Abstract

Amphibious plants, living in land-water ecotones, have to cope with challenging and continuously changing growth conditions in their habitats with respect to nutrient and light availability. They have thus evolved a variety of mechanisms to tolerate and adapt to these changes. Therefore, the study of these plants is a major area of ecophysiology and environmental ecological research. However, our understanding of their capacity for physiological adaptation and tolerance remains limited and requires systemic approaches for comprehensive analyses. To this end, in this study, we have conducted a mesocosm experiment to analyze the response of *Butomus umbellatus*, a common amphibious species in Denmark, to nutrient enrichment and shading. Our study follows a systematic integration of morphological (including plant height, leaf number, and biomass accumulation), ecophysiological (photosynthesis-irradiance responses, leaf pigment content, and C and N content in plant organs), and leaf metabolomic measurements using gas chromatography-mass spectrometry (39 mainly primary metabolites), based on bioinformatic methods. No studies of this type have been previously reported for this plant species. We observed that *B. umbellatus* responds to nutrient enrichment and light reduction through different mechanisms and were able to identify its nutrient enrichment acclimation threshold within the applied nutrient gradient. Up to that threshold, the morpho-physiological response to nutrient enrichment was profound, indicating fast-growing trends (higher growth rates and biomass accumulation), but only few parameters changed significantly from light to shade [specific leaf area (SLA); quantum yield (*φ*)]. Metabolomic analysis supported the morpho-physiological results regarding nutrient overloading, indicating also subtle changes due to shading not directly apparent in the other measurements. The combined profile analysis revealed leaf metabolite and morpho-physiological parameter associations. In this context, leaf lactate, currently of uncertain role in higher plants, emerged as a shading acclimation biomarker, along with SLA and *φ*. The study enhances both the ecophysiology methodological toolbox and our knowledge of the adaptive capacity of amphibious species. It demonstrates that the educated combination of physiological with metabolomic measurements using bioinformatic approaches is a promising approach for ecophysiology research, enabling the elucidation of discriminatory metabolic shifts to be used for early diagnosis and even prognosis of natural ecosystem responses to climate change.

## Introduction

The land-water ecotone is a highly abundant ecosystem of substantial ecological value but, at the same time, one of the most threatened habitats worldwide (Strayer and Findlay, 2010). This highly dynamic and diverse ecosystem (Naiman and Décamps, 1997; Ward et al., 2002) is associated with different plant life forms, with amphibious species occurring both on land and submerged in the streams. Amphibious plants have evolved a variety of survival mechanisms to withstand sudden emersion and submersion and cope with continuously changing environmental conditions caused by fluctuating water levels, temperature, light availability, and nutrients (Maberly and Spence, 1989). They are usually submerged in shallow water close to the bank, probably because their permanent bank populations can disperse to the water by ingrowth (Henry and Amoros, 1996; Riis et al., 2001) and thus, canopy shading can reduce light attenuation in forested riverbanks. Light limitation becomes more profound for amphibious plants living in waters with high nutrient levels, since dense populations of both biofilms and phytoplankton lead to lower light penetration to leaf surfaces (Sand-Jensen and Borum, 1991; Lassen et al., 1997).

Hydromorphological degradation, caused by agricultural practices and channelization, has substantially influenced the natural distribution of the land-water ecotone of lowland streams in Europe (e.g., Mattingly et al., 1993; Verdonschot and Nijboer, 2002; Landwehr and Rhoads, 2003). In Denmark, the land-water ecotone has been significantly reduced over the last 200 years (Madsen, 1995), with subsequent restriction of ecological niches available for amphibious plants (Baattrup-Pedersen et al., 2016). Understanding the mechanisms behind amphibious species distribution and diversity is very important for stream management and restoration under various environmental challenges, and the implementation of appropriate protection measures for freshwater biodiversity.

*Butomus umbellatus* is a characteristic amphibious species of the land-water ecotone, and among the dominant species in large lowland streams in Denmark (Riis et al., 2001). Thereby, it can be used as a model to study the land-water ecotone changes in these ecosystems. The species has two types with different ploidities (diploid and triploid), and there have been numerous studies focusing on their differences in reproduction (Fernando and Cass, 1997; Bhardwaj and Eckert, 2001), ecology (i.e., Harms, 2020), growth (Hroudová et al., 1996), and on factors related to water level fluctuations. *B. umbellatus* morphological traits, mainly its well-developed rhizomes and the basal meristem, permit its survival in eutrophic sites (Baattrup-Pedersen et al., 2015), allowing regrowth after weed cutting. However, no comprehensive ecophysiological information on its response to eutrophication and light limitation is yet available, thus current knowledge regarding its adaptive capacity to environmentally challenged habitats is limited.

Environmental ecology and ecophysiology research can benefit from the use of high-throughput biomolecular (omic) analyses to extend the current methodological toolbox and obtain an enhanced perspective of the adaptability and survival mechanisms of the amphibious plants under various environmental stresses. Metabolomics in particular, targeting the highly dynamic metabolic physiology, can be used to identify and quantify species-specific ecophysiological adjustments at the metabolic level, linking the genetic background to environmental stresses. The application of metabolomics in ecology (eco-metabolomics) promises to reveal the biochemical basis of various ecological responses (Peters et al., 2018), enriching the measurements of the plant ecological phenotype with molecular data (van Dam and van der Meijden, 2011).

In this ecological and technological context, we studied the *B. umbellatus* response to nutrient enrichment and shading conditions, in a mesocosm experiment. Plant response characterization was based on the integration of ecophysiological, phenotypic, and leaf metabolomic measurements, using a bioinformatic approach. We hypothesized that in order to endure different combinations of environmental settings, plants would elicit unique acclimation strategies through specific alterations of their energy balance, carbon assimilation, and growth rate, and these changes will be reflected in their metabolic physiology. Main questions that were investigated through this study are:

- Do individuals grown in different treatments exhibit distinctly differential morphological and photosynthetic responses?
- How is leaf metabolic profile correlated with morpho-physiological responses at the various treatments?
- Are there any eco-physiological factors or metabolite abundances individually or in combination that can be considered as shade-specific or nutrient-specific biomarkers?

## Materials and Methods

### Mesocosm Set-up

The study was conducted at the experimental field station Påskehøjgaard, 10 km north of Aarhus, Denmark, during summer 2018. The mesocosm design comprised 30 partially buried 90 L, 60 cm (diameter) × 33 cm (height), round plastic tubs. Each mesocosm was filled up with 24 L of washed beach sand sediment and 52 L tap water. Each tub was oxygenated with atmospheric air and provided with 1.2 L tap water per hour through a drip tube (2-day retention time).

All individuals of *B. umbellatus* were collected from the same population from Gudenå stream in East Jutland, Denmark, in June 2018. Apical shoots were shortened to 15 cm and placed in aquariums (27 L) containing tap water and oxygen tubes. The shoots were then acclimated in experimental conditions of 20°C mean daily air temperature and 1,02 (μmol m^−2^ s^−1^) mean daily photosynthetic active radiation (PAR) for 3 days before planting.

### Applied Treatments

The experiment ran for 11 weeks (June–September 2018) and involved the investigation of plant response to changes in nutrient enrichment and/or shading. Five nutrient concentrations (NL1–NL5), in two light availability conditions (full exposure to sunlight, “Open;” and 50% shading, by a shade-cloth mounted over the tub, “Shade”), were established in triplicate, totaling 10 treatments and 30 mesocosm tubs. Nutrient level (NL) 1 was the baseline nutrient composition, having been created from 24 L of washed beach sand (sediment) and 52 L Aarhus Municipality tap water without the addition of commercial fertilizer slow release pellets (NPK Macro 15-5-11 and Micro with iron, Osmocote Plus, Denmark). In the other treatments, commercial fertilizer pellets were added to the “baseline” water: 10, 20, 40, and 60 pellets, respectively for NL2, NL3, NL4, and NL5 (Table 1). The assigned nutrient level in each tub was maintained constant through pellet addition every 7–10 days. Every 7 days, mesocosms were cleaned from any macroscopic green algae using a brush.

**Table 1:** Nutrient composition based on the number of commercial fertilizer pellets used at the five nutrient levels examined in the study.

Fertilizer nutrient	NL 1 [1] (0 fertilizer pellet) [2]	NL 2 (10 fertilizer pellets; g)	NL 3 (20 fertilizer pellets; g)	NL 4 (40 fertilizer pellets; g)	NL 5 (60 fertilizer pellets; g)
Nitrogen (N)	-	11.25	22.50	45.00	67.50
Phosphorus (P)	-	3.60	7.20	14.40	21.60
Potassium (K)	-	8.10	16.20	32.40	48.60
Magnesium (Mg)	-	0.90	1.80	3.60	5.40
Boron (Bo)	-	0.15	0.30	0.60	0.90
Copper (Cu)	-	0.38	0.75	1.50	2.25
Iron (Fe)	-	0.30	0.60	1.20	1.80
Manganese (Mn)	-	0.45	0.90	1.80	2.70
Molybdenum (Mo)	-	0.15	0.30	0.60	0.90
Zink (Zn)	-	0.11	0.23	0.45	0.68

[1] Baseline = Aarhus Municipality tap water (*N* = 0.89 mg/l; Mg = 8.4 mg/l; Bo = 63 μg/l; Cu = 8.8 μg/l; Fe < 0.01 mg/l; Mn = <0.002 mg/l; Zn = 1.3 μg/l); Data from Aarhus Municipality tap water from https://www.aarhusvand.dk. [2] 1 pellet contains nitrogen: 1.125 g; phosphorus: 0.36 g; potassium: 0.81 g; magnesium: 0.09 g; boron: 0.015 g; copper: 0.0375 g; iron: 0.03 g; manganese: 0.045 g; molybdenum:0.015 g; zink: 0.01125 g; n -0.110 230.45 0.81 pellet contains nitrogen: 1.125 g; phosphorus: 0.36 g; potassium: 0.81 g; magnesium: 0.09 g; boron: 0.015 g; copper: 0.0375 g; iron: 0.03 g; manganese: 0.045 g; molybdenum:0.015 g; zink: 0.01125 g.

We opted for the use of slow release pellets to create the eutrophication gradient, instead of the addition of nutrient solution in the water, because *B. umbellatus* takes up nutrients from the soil, and we wanted to imitate the natural conditions. In addition, the use of pellets is preferable to avoid opportunistic algae growth in the water. The existence of the eutrophication gradient in our experiment was confirmed by the internal tissue *N* (see Supplementary Figure S1; Gusewell and Koerselman, 2002). The selection of the number of pellets per treatment was based on initial laboratory experiments. The release of nutrients to the water as indicated by the water chemical analyses showed that a significant increase in the number of pellets per treatment was required to create a nutrient gradient.

The average daily and maximum PAR during the experiment for the open treatment were 602 and 2029 μmol m^−2^, respectively (Supplementary Figure S2). PAR was measured at a nearby climate station, and the shading conditions were tested with a handhold light meter.

### Photosynthetic Activity Measurements

At the end of the experiment, one photosynthetic gas exchange measurement per replicate was made on a fully developed leaf (5 cm from the apex), using an infrared gas analyzer (IRGA) model 6400XT (LiCor Lincoln NE, United States), with a 6400-02B red/blue LED light source (chamber temperature = 16°C; relative humidity = 60–90%; CO_2_ = 400 ppm). The leaf area inserted in the chamber was measured with a ruler prior to the analysis. The leaves were acclimated in the chamber for at least 3 min at a light intensity of 2,000 μmol m^−2^ s^−1^ until steady-state gas exchange rates were achieved, and the measurements were recorded using the IRGA light curve program. The measurements were made at the following light intensities (minimum wait time: 60 s, maximum wait time: 180 s) in this order: 2,000, 1,700, 1,500, 1,000, 700, 500, 250, 100, 75, 25, and 0 μmol m^−2^ s^−1^. Using the tool of de Lobo et al. (2013), we chose the light-response curves of the best fit model (Baly, 1935), and the photosynthetic parameters were estimated from the Michaelis-Menten based model (Supplementary Table S1).

After the photosynthesis readings, the leaf in the chamber was cut, weighed, frozen, and freeze-dried for C and N analyses. An extra leaf was collected from each plant just below the part in the chamber for pigment measurements.

### Plant Growth and Biomass Allocation Measurements

Plants were removed, transported to the laboratory in cooling boxes rinsed with ultra-purified water, and blotted with tissue paper to remove moisture before weighing [fresh weight (FW)]. They were separated into below‐ and above-ground parts. After counting leaf number and measuring the above-ground height, both parts were weighed, frozen, and freeze-dried to determine their dry weights (DWs). The relative growth rate (RGR) was estimated based on the mean of natural logarithm transformed DWs, according to Hoffmann and Poorter (2002) (FW/DW = 20.31, see also Supplementary Table S1 for details).

### Leaf Components and Pigments: C/N Organ Content Analyses

Chlorophyll *a, b* and carotenoid (carotenes and xanthophylls) concentrations in the leaves were quantified following the work of Lichtenthaler (1987), using a UV-VIS spectrophotometer (SHIMADZU).

Organ (leaf, shoot, and root) content (% of DW) in carbon (C) and nitrogen (N) were determined for the IRGA chamber leaf and the below (roots) and above (shoot) – ground parts of each plant, from a 2–5 mg powdered sample, using a C/N analyzer (Elementar Vario El Cube). The specific leaf area (SLA) was estimated from the ratio of the IRGA chamber leaf area with its DW. Photosynthetic nitrogen use efficiency (PNUE) was calculated according to Hidaka and Kitayama (2009) (Supplementary Table S1).

The complete list of ecological and eco-physiological measurements with the respective acquisition method(s) are shown in Supplementary Table S1.

### Metabolomic Analysis

Metabolomic analysis was performed for NL1 and the high nutrient concentrations NL4 and NL5, which simulate eutrophic conditions, at both open and shade treatments, to identify relevant to high nutrient enrichment metabolic activity shifts. Three fully developed, mature leaves per mesocosm were collected directly after the photosynthesis measurements, rapidly rinsed with tap and then deionized water, frozen in liquid nitrogen, and stored at −80°C. They were transferred in dry ice to the laboratory performing untargeted GC-MS metabolomic analysis. The leaves of each mesocosm were pooled and grounded to powder with mortar and pestle in liquid nitrogen. A hundred (100) mg of leaf powder from each mesocosm was used for the polar metabolite extraction based on the methanol/water protocol described by Dutta et al. (2009), Kanani et al. (2010), and Tooulakou et al. (2016), after adding 10 μg ribitol (AlfaAesar, Germany) and 20 μg (U-^13^C)-glucose as internal standards (Cambridge Isotope Laboratories, USA).

The metabolic profile acquisition procedure is described by Papadimitropoulos et al. (2018). The metabolic profile of each sample was measured at least thrice. The peak identification and quantification were based on the commercial NIST and in-house peak library (Maga-Nteve and Klapa, 2016; Papadimitropoulos et al., 2018). The metabolic profile data validation, normalization, and filtering were carried out using the M-IOLITE software suite (http://miolite2.iceht.forth.gr; Maga-Nteve and Klapa, 2016), estimating the marker ion relative peak area (RPA) of each metabolite derivative with respect to the peak area of ribitol ion 217. Finally, the normalized and filtered profiles comprised 39 metabolites. The metabolic profile of each mesocosm was estimated as the mean of the normalized profiles of its technical replicates, and then the metabolic profile of each physiological state was estimated as the mean of the normalized profiles of its three biological replicates/mesocosms. The final normalized dataset used in further analysis is provided in Supplementary Table S2.

### Statistical Analysis

The experiment was a two-factorial design with “light regime” and “nutrient level.” All variables were tested for normality and homoscedasticity prior to statistical analysis using the Shapiro–Wilk normality test (function shapiro.test in the R package stats). Two-way ANOVA (Mangiafico, 2015) was performed for each morphological and physiological measurement to test whether it was significantly affected by any of the two factors and/or their interaction at a significance threshold *p* < 0.05. These statistical analyses and visualization were carried out in R version 3.5.3 (R Core Team, 2019).

Metabolic profiles individually or combined with the morpho-physiological measurements were analyzed using the multivariate statistical analysis algorithms of hierarchical clustering (HCL), *k*-means clustering, principal component analysis (PCA), and significance analysis for microarrays (SAM), as implemented in the open source omic data analysis TM4 MeV software (version 4.9.0; Saeed et al., 2003, 2006). The analyses were performed on the standardized metabolic profiles, in which the standardized RPA of metabolite *M* in profile *j*, $stRPA_{M}^{j}$, is estimated as follows:

$$stRPA_{M}^{j}=\frac{RPA_{M}^{j}-\overset{-}{RPA_{M}}}{SD_{RPA_{M}}}$$

where $RPA_{M}^{j},\overset{-}{RPA_{M}},SD_{RPA_{M}}$ depict, respectively, the RPA of metabolite M in profile j, the mean RPA of metabolite M, and its standard deviation in all profiles. The metabolites, the concentration of which was identified as significantly higher or lower in a set of metabolic profiles compared to another, will be, respectively, referred to as positively or negatively significant metabolites of the particular comparison.

## Results

### Plant Growth and Biomass Allocation

**Figure 1:**
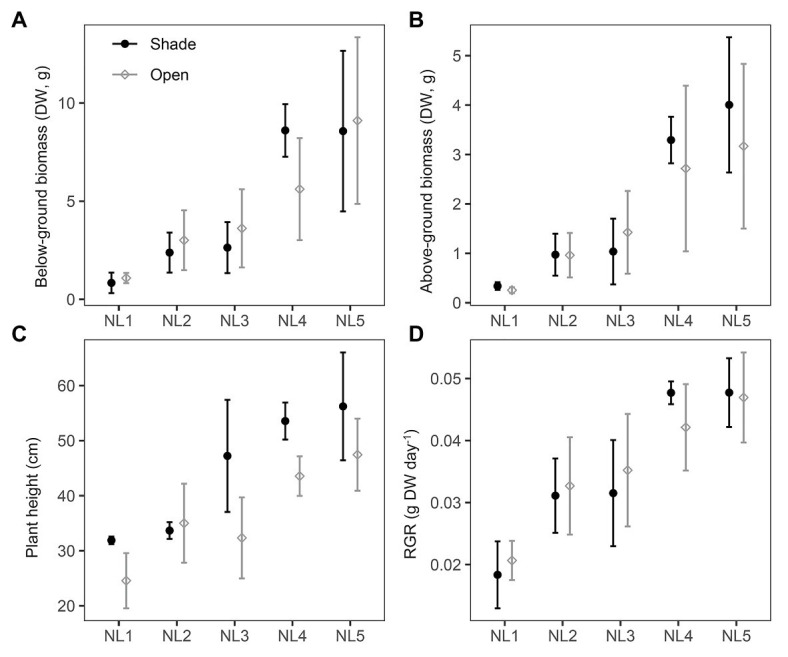
Scatter (XY) plots plots with standard deviation (±SD) of the mean **(A)** below-ground biomass and **(B)** above-ground biomass, **(C)** plant height, and **(D)** relative growth rate (RGR) at the various nutrient levels and light regimes. NL, nutrient level; DW, dry weight. Filled black circles, shade treatments; gray open circles, open treatments. SD estimated over *n* = 3 mesocosms per treatment.

All individuals survived under the experimental conditions. The production of both above‐ and below-ground biomass was significantly affected by nutrient enrichment, while no significant changes occurred between open and shade treatments (Table 2). Biomass production of the individuals grown in the open mesocosms followed a continuously increasing trend from low to high nutrient availability (Figures 1A,B). Below-ground biomass of shade individuals reached its highest value at NL4 treatment with no further significant change in NL5 (Figure 1A). Both nutrient levels and light availability affected the final plant height with an increase from NL1 to NL5. Overall, the height of the individuals from the shade treatments was significantly higher compared to the height of plants from the open mesocosms (Figure 1C). RGR was significantly affected by the nutrient enrichment, but not by the light reduction (Table 2). The highest RGR values were measured in high nutrient level treatments (SH-NL4, SH-NL5, and OPEN-NL5; Figure 1D; Supplementary Table S3).

**Table 2:** Results of two-way ANOVA of morpho-physiological parameters.

Nutrient related parameters	F-factor	Response to nutrients
Above ground biomass (DW; g)	12.897^***^	↑
Below ground biomass (DW; g)	12.334^***^	↑
Number of leaves	10.171^***^	↑
RGR (g DW day^−1^)	17.587^***^	↑
Chl *a*:*b*	9.534^***^	↑
%C per shoot DW	5.714^***^	↑
C:N in root	16.834^***^	↓
Ik [μmol (photons) m^−2^ s^−1^]	9.149^***^	↑
C:N in shoot	20.542^***^	↓
PNUΕ (μmolCO_2_/gN/s)	5.640^**^	↓
%N per leaf DW	4.388^**^	↑
%C per leaf DW	2.961^**^	↑
P_max_ [μmol (CO_2_) m^−2^ s^−1^]	3.875^*^	↑
Rd [μmol (CO_2_) m^−2^ s^−1^]	3.023^*^	↓
C:N_leaf	4.2^*^	↓
**Shade related parameters**	**F-factor**	**Response to Shade**
SLA (cm^2^ g^−1^)	5.836^**^	↑
*φ* [mmol (CO_2_) mmol^−1^ (photons)]	5.029^*^	↑
**Nutrient and shade related parameters**	**F-factor**	**Response to nutrient and shade**
Plant height (PH; cm)	14.347^***^ (N) 11.800^**^ (S)	↑ ↑
%N per root DW	11.463^***^ (N) 6.046^**^ (S)	↑ ↑
%N per shoot DW	14.537^***^ (N) 4.185^*^ (S)	↑ ↑
Carotenoids (mg DWg^−1^)	10.979^***^ (N) 7.771^*^ (S)	↑ ↑
Chl-*a* (mg DWg^−1^)	13.918^***^ (N) 32.315^***^ (S)	↑ ↑

Only parameters that changed significantly with either nutrient enrichment (N) or shading (S) or their interaction are shown (↑: increase; ↓: decrease; DW, dry weight).

* Level of significance *p* < 0.05.

** Level of significance *p* < 0.01.

*** Level of significance *p* < 0.001.

### Specific Leaf Area and Pigments

Specific leaf area was 17% higher in the shade compared to the open mesocosms (shade mean: 181 cm^2^ g^−1^; open mean: 155 cm^2^ g^−1^; with the highest value 197 cm^2^ g^−1^ at SH-NL3; Table 2; Supplementary Table S4). Both light regime and nutrient level associated changes were observed in the photosynthetic pigment composition (Table 2). Chl-*a* and carotenoid concentrations were significantly higher in the shade than in the open mesocosms (Table 2; Supplementary Table S4), while Chl *a*:*b* was found to be significantly higher in high nutrient level treatments regardless of the light conditions.

### Organ Nitrogen and Carbon Concentration

Root and shoot N concentrations were affected by both light regime and nutrient level. Root *N* doubled from NL1 to NL5 for both open and shade treatments. This change was much higher for Shoot N, which was of almost three times higher abundance in NL5 compared to NL1 both in the open and shade mesocosms (Supplementary Table S3). C concentration showed a small increase in the leaves (7 and 9% in shade and open treatments, respectively) and the shoot (14 and 16% in shade and open treatments, respectively) with the nutrient level, with no significant change observed in the root in either treatment.

The C:N ratio revealed intrinsic differences in the allocation of C and N to leaves, shoots, and roots due to differences in nutrient level between the open and shade treatments (Table 2), with the N allocation to leaves being in all cases higher than to the other organs (Supplementary Table S3). Nutrient level affected also PNUE, which was observed decreasing by up to 2-fold in the leaves of *B. umbellatus* grown at NL4 and NL5 compared to NL1 (Supplementary Table S3).

### Photosynthetic Light-Response

**Figure 2:**
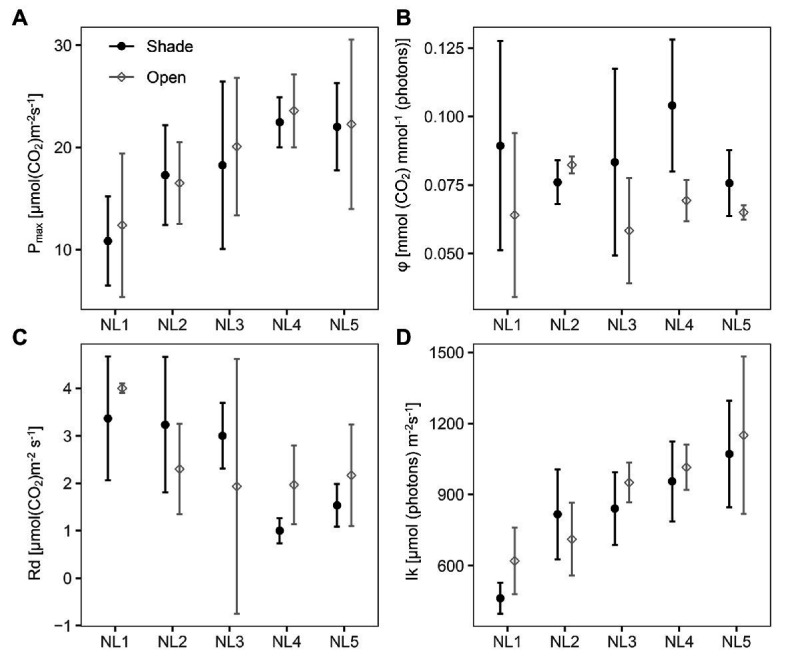
Scatter (XY) plots plots with standard deviation (± SD) of the mean **(A)** light-saturated photosynthesis rate, P_max_; **(B)** quantum yield, φ; **(C)** dark respiration, Rd; and **(D)** light saturation point, Ik at the various nutrient levels and light regimes; NL, nutrient level. Black filled circles, shade treatments; gray open circles, open treatments. SD estimated over *n* = 3 mesocosms per treatment.

The fitted photosynthetic-light response curves for all treatments are presented in Supplementary Figure S3. The light-saturated photosynthesis rate (P_max_), dark respiration (Rd), and the light saturation point (Ik) were significantly affected by the nutrient level with no difference between the two light regimes, while the quantum yield (φ) did not change between nutrient levels but was significantly higher in the shade treatments (Figure 2; Table 2). The light compensation point (Ic) showed a decreasing trend with the nutrient enrichment, reaching, however, a plateau at NL4 with no significantly different value at NL5. The values of Ic between open and shaded treatments of the same nutrient level were similar (Supplementary Figure S1).

P_max_ presented on a leaf area basis increased gradually with the nutrient level up to NL4 with no apparent change in NL5, reaching a double value in NL4 and NL5 compared to NL1, with Rd exhibiting the opposite – decreasing – trend with nutrient enrichment (Figure 2). Ik exhibited a linear increase with nutrient enrichment [Shade mean: 829 μmol (photons) m^−2^ s^−1^; open mean: 889 μmol (photons) m^−2^ s^−1^; with highest mean value 1,151 μmol (photons) m^−2^ s^−1^, at OPEN-NL5; Table 2; Supplementary Table S4].

### Leaf Metabolomic Analysis

**Figure 3:**
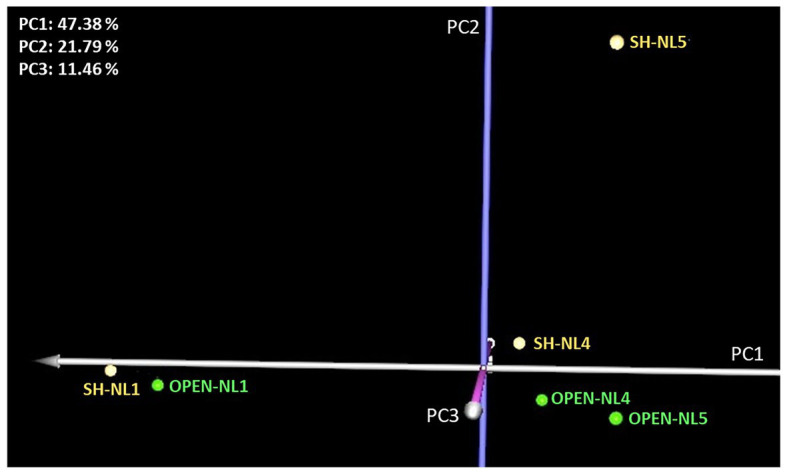
Principal component analysis (PCA) graph of the standardized leaf metabolic profiles at NL1, NL4, and NL5 for both light regimes. A large difference is observed in the metabolic profiles of NL4 and NL5 with respect to NL1 in both light regimes (manifested on PC1 axis), while the distinction between the profiles of the shade and open treated samples is apparent on PC2.

Leaf metabolic profiling was applied to the NL1, NL4, and NL5 treatments in both light regimes, to investigate whether nutrient overloading symptoms could be apparent at the molecular level before becoming clearly observable in the eco-physiological measurements. PCA analysis indicated a substantial difference between the NL4 and NL5 profiles compared to the NL1 profile in both light regimes, manifested on PC1 axis (Figure 3). In addition, there is a distinction in the profiles between the two light regimes, manifested on PC2, with the difference increasing with the nutrient level, becoming substantially large at NL5.

Hierarchical clustering analysis (HCL) supported the PCA findings (Figure 3) also revealing metabolite clusters based on their concentration profile in the various treatments (Supplementary Figure S4). One notable cluster comprises the metabolites exhibiting a largely different abundance in SH-NL5 compared to the other treatments. It is the change in these metabolites that mainly characterized the substantial difference observed in the leaf metabolic profile of the plants under these conditions (Figure 3; Supplementary Table S2; Supplementary Figure S4). These metabolites are glycerate, fumarate, malate, glutamate, linoleic acid, ethanolamine, serine, phytol, phosphate, and three unknowns including a sugar (increased abundance in SH-NL5), and the unknown sugar, Un_240 (decreased abundance in SH-NL5).

To identify the metabolites that are markers of the shade conditions characterizing the difference in the metabolic profiles of the same nutrient level between the two light regimes over PC2 (Figure 3), we performed multivariate significance analysis based on the SAM method, considering all open treatment profiles at any nutrient level as Group A and all shade treatment profiles at any nutrient level as Group B. Lactate, serine, phytol, glycerol, and phosphate were identified as positively significant (in decreasing order of significance) and Un_240 as negatively significant, in the shade vs. the open conditions (Supplementary Figures S5, S6). Lactate and glycerol, along with two unknowns, P1188 and Un_0236, were also identified as statistically significant when paired-SAM was applied, which takes into consideration the nutrient level effect by pairwise comparisons of the shade vs. the open treatment samples at the different levels. Hence, lactate was identified as marker of shade conditions independently of the nutrient level. It was also observed that the extent of lactate increase due to shading decreases with the nutrient enrichment. Interestingly, the abundance of sugar Un_240 exhibits an opposite profile, with the extent of its decrease due to shading increasing from NL1 to NL5 (Supplementary Figure S6).

### Integrated Multivariate Analysis of the Metabolomic With the Morpho-Physiological Data

An objective of the present study was to search for clusters of metabolites and morpho-physiological measurements exhibiting similar patterns over the various treatments, linking thus the molecular with the “classical” physiological/phenotypic data in an effort to study them comprehensively. Therefore, we combined all measurements of each physiological state into an integrated phenomic profile and analyzed the dataset of all treatments using multivariate statistical analysis. The PCA graph of the combined profiles (Supplementary Figure S7) was similar to that of the metabolic profiles (Figure 3), indicating however a larger difference between OPEN-NL4 and OPEN-NL5 conditions and between the open and shade conditions at each respective nutrient level. The combined profile analysis also suggested a large physiological difference of SH-NL5 treated plants compared to the other mesocosms.

**Figure 4:**
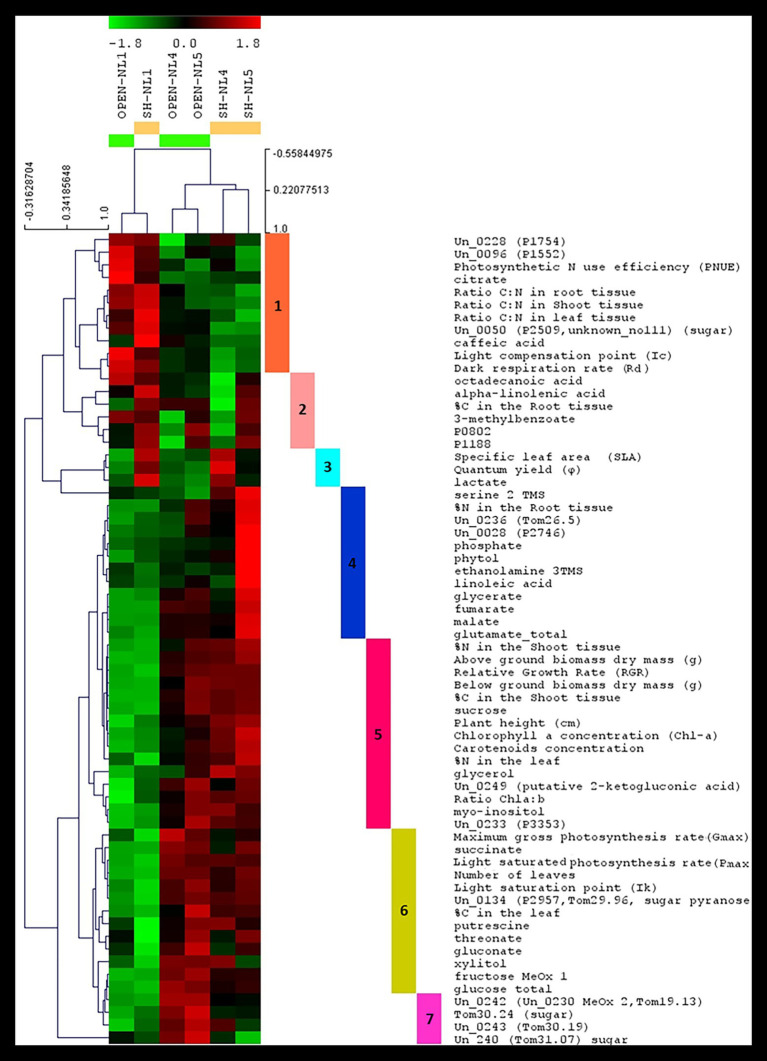
Hierarchical clustering analysis (HCL) heat map of the standardized leaf combined profiles at NL1, NL4, and NL5 for both light regimes. The colored vertical bars indicate clusters of parameters/metabolites with similar profiles over the investigated treatments. Pearson correlation coefficient distance metric was used. Negative (colored in shades of green) or positive (colored in shades of red) standardized value indicates that a parameter/metabolite is of lower or higher, respectively, value/abundance at the particular treatment compared to its mean value over all treatments. The image has been created in and saved from TM4 MeV software.

**Figure 5:**
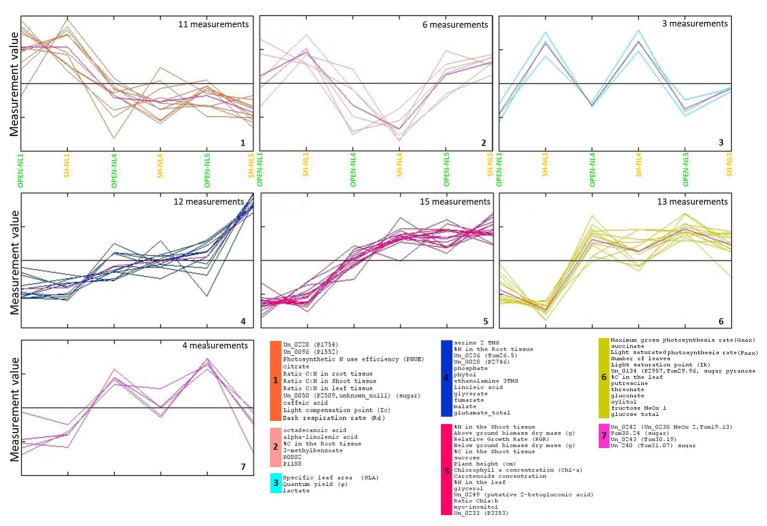
Τhe clusters of morpho-physiological factors and metabolites identified by *k*-means clustering of the standardized combined profiles at NL1, NL4, and NL5 for both light regimes.. The mean profile in every cluster is shown in purple. Euclidean distance metric was used. Cluster numbers and colors refer to the corresponding HCL-identified in Figure 4. The image has been created in and saved from TM4 MeV software.

HCL and *k*-means clustering of the combined profiles revealed clusters of morpho-physiological parameters and metabolite abundances exhibiting similar profiles over the various treatments (Figures 4, 5). The phenomic clusters are divided into two main categories: those exhibiting a higher value at NL1 (open and/or shade conditions) over the other two nutrient levels (first three clusters in Figures 4, 5; 1: orange, 2: light pink, and 3: light blue) and those with a higher value at NL4/NL5 over NL1 (last four clusters in Figures 4, 5; 4: blue, 5: pink, 6: yellow, and 7: magenta). There are more morpho-physiological parameters and metabolites in the second than in the first category. The profile characteristics of each cluster within the two main categories over the various treatments are described below:

- The orange (1) cluster includes factors that decrease with the nutrient enrichment, but are not largely affected by the shade treatment (PNUE, Ic, C:N ratio in root, leaf and shoot, Rd, citrate and caffeic acid among the known metabolites). They reach a plateau at NL4, exhibiting similar values at NL5.
- The factors in the pink (5) cluster exhibit the opposite trend. These include the plant height, below‐ and above-ground dry biomass, RGR, Chl-*a* and carotenoid concentration, Chl *a*:*b*, %N in shoot and leaf, % C in shoot and sucrose, myo-inositol, glycerol, and 2-ketogluconic acid (putative) among the known metabolites.
- The factors in the yellow (6) and magenta (7) clusters are of similar profile, increasing with the nutrient enrichment in the open treatments with a plateau at NL4, being, however, also negatively affected by the shading, especially at the high nutrient levels. The yellow cluster factors, including sugars (glucose and fructose), sugar alcohols (xylitol), sugar acids (threonate and gluconate), succinate, and putrescine along with % leaf C, Ik, P_max_, and leaf number, show similar response to shading independently of the nutrient level, while for those in the magenta cluster (i.e., four unknown metabolites, with Un_240, Un_0242, Tom30.24 being putative sugars/sugar acids), the negative shading effect increases with the nutrient enrichment.
- The blue cluster (4) includes the factors characterizing the substantial difference in the combined profiles of the shade-NL5 treated plants from all others. These factors exhibit a substantial increase in shade-NL5 compared to open-NL5 and include the metabolites identified as characteristic of this shade-NL5 difference in metabolic profiling analysis and the % root *N*.
- The factors in the light pink (2) cluster, including the fatty acids octadecanoate (stearate) and alpha-linolenic acid, 3-methylbenzoate, unknowns P0802 and P1188, and the % root C, exhibit a minimum at (shade) NL4 conditions, increasing at NL5 treatments.
- Finally, this analysis identified lactate as tightly clustering with the shading biomarkers SLA and φ; light blue cluster (3).

**Figure 6:**
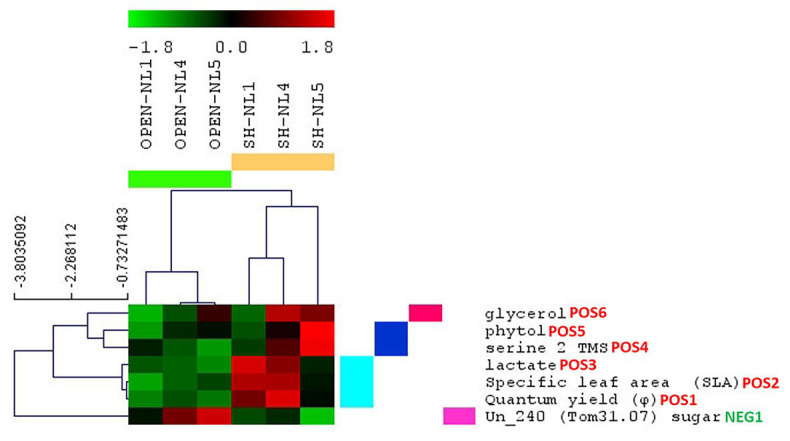
Morpho-physiological parameters and metabolite abundances identified as significantly changing in the shade versus the open treatments. Results are based on significance analysis for microarrays (SAM), and the parameters are clustered based on their profile through all treatments. The indication POS or NEG next to the name of each parameter/metabolite differentiates, respectively, the positively from the negatively significant in the shade compared to the open treatments; the number depicts the significance hierarchy of each parameter/metabolite in its respective group..

To identify the morpho-physiological factors and metabolites that are affected significantly by the shading, we applied SAM, considering the combined profiles of the open treatments into Group A and those of shade treatments into Group B. We validated as positively significant in shade vs. open treatments, the φ, SLA, lactate cluster, and the metabolites serine, phytol and glycerol, as in the respective analysis of the metabolomic dataset, with Un_240 (sugar) being the only negatively significant metabolite (Figure 6). Paired-SAM comparing open and shade treatments in the NL pairs identified also plant height (as the most significant), carotenoid concentration, Un_0236, P1188 and Chl-*a* as positively significant in shade vs. open treatments, but not serine, which did not pass the significance threshold in this analysis. As negatively significant in shade, paired-SAM identified gluconate, Rd, Ic, and G_max_, in this order of significance.

## Discussion

In this study, we performed a comprehensive eco-physiological investigation combined with metabolomic analysis of the amphibious *B. umbellatus* plant response to nutrient availability changes and shading, in a mesocosm experimental set-up. This is the first ecophysiological study of that scale for the effect of the two factors in this species. The selection of the experimental conditions enabled us to investigate the physiological effect of nutrient increase in the growth environment of amphibious plants, with or without shading, up to excess nutrient levels, simulating thus gradually more challenging habitats. The measurements of the present study provide a combined perspective of the phenotype and molecular physiology of *B. umbellatus* under demanding conditions, enabling the determination of nutrient saturation and physiological turning points to be further validated by field experiments. Moreover, combined with leaf metabolic profiling, this type of study is methodologically unique for amphibious plants and represents a novel perspective for all plant ecophysiological studies in general.

### Combined Metabolic and Ecophysiological Profiling Revealed NL4 as Nutrient Overloading Threshold

The experimental design, by investigating the effect of nutrient enrichment and shading individually and in combination, enabled the identification of excess nutrient conditions for *B. umbellatus*, where the plant was observed to be reaching its limits for physiological acclimation and tolerance. This is a notable result as it adds to the current knowledge about the particular species and underlines the significance of carrying out such type of ecophysiological studies to define the tolerance threshold of a plant to various environmental stresses that can be encountered in its habitats. More specifically, NL4 was revealed as the nutrient-enrichment saturation threshold in this study by a combination of findings and observations. Most measurements level-off at NL4 in open-treatment conditions (Figure 5). Nutrient enrichment up to NL4 was associated with higher RGR, biomass accumulation (both below‐ and above-ground), higher number of leaves, larger % leaf N and C, % shoot C, and higher concentrations of: sugars (sucrose, glucose, and fructose) and glycerate, glutamate, a significant molecule for plant nitrogen metabolism (Brian et al., 2007), the tricarboxylic cycle intermediates, succinate, fumarate, malate, the polyunsaturated fatty acid (PUFA) linoleic acid, 2-ketogluconic acid, and glycerol (Figures 1, 2, 4, 5). To accommodate the higher carbon requirements, plants had to increase their photosynthetic rate and photosynthetic organs (number of leaves), which lead to the observed decrease in the leaf C:N ratio, dark respiration and photosynthetic N use efficiency (PNUE). These changes are expected, as higher growth rates mean higher demand for carbon, and thus higher leaf N content and lower C:N ratio (e.g., Chapin et al., 1993).

Our measurements indicate a more efficient photosynthesis in lower nutrient availability conditions (Eller and Brix, 2012), while the “plateau” observed in these measurements at NL4 to NL5 imply that the plant has been reaching its threshold of nutrient enrichment acclimation based on its available genetic predisposition. Considering this state of “saturation” as a state of “unstable equilibrium” for the plant, which has reached a “turning point” in accommodating its growth with the particular nutrient availability, any additional (even moderate) negative event/stress is expected to have a much larger impact on the plant metabolic physiology than in lower nutrient availability conditions. It is in this context that the significantly different metabolic and combined profile of the plant at SH-NL5 compared to SH-NL4 conditions has been interpreted (Figure 3; Supplementary Figure S7). Shading is considered a negative event for the plant as lowering the light availability and thus, the photosynthetic capacity of the plant. While the particular shading applied in this study does not cause a large difference in the overall metabolic physiology of the plants at NL1 and NL4, its effect is dramatic at NL5. This observation supports NL4 as the nutrient enrichment acclimation threshold for the plant, which becomes apparent with the combined application of the shading regime (combined effect and interaction). Stress conditions are implied by the metabolite abundances/physiological measurements, which increase dramatically between SH-NL4 and SH-NL5 and are characteristic of the observed change in the metabolic and combined profiles of the plants between these two conditions (Figures 3, 5; Supplementary Figure S7). Apart from the known correlation between the N content and the photosynthetic efficiency (P_max_) through the investment of N in compounds involved in photosynthesis (Vos et al., 2005; Eller and Brix, 2012; Mathur et al., 2018), the combined profile analysis indicated a direct connection of the C and N content of the root with certain leaf metabolite abundances. Increase of %N in the roots might be correlated with increased assimilation of N by the roots to transfer it to the chloroplasts, where it is transformed into glutamate (Lea and Forde, 1994). The observed increase in the particular intermediates of the glycerate, glutamate, and the tricarboxylic acid (TCA) cycle intermediates fumarate and malate, combined with the increase in the fatty acids suggest higher need for anaplerosis of the TCA cycle through a higher activity of the glutamate anaplerotic pathway and a potentially higher rate of lipid oxidation through the glyoxylate cycle. Such measures suggest oxidative stress conditions for the plants, further supported by the implied increase in the activity of serine metabolism and glycerate/serine non phosphorylated pathway (Igamberdiev and Eprintsev, 2016). It is noted that alpha-linolenic and stearic acids are precursors of jasmonic acid, a phytohormone important for the mediation of stress response (Ruan et al., 2019).

### Metabolic and Combined Profiles Are Discriminatory of Shading Acclimation Changes Independently of the Nutrient Level

The multivariate statistical analysis of the metabolic and combined profiles revealed changes caused by shading independently of the nutrient level (Figures 3, 6; Supplementary Figures S5, S7). This result supports the need to apply multivariate analyses in omic and morpho-physiological measurements, which are interconnected and interdependent. Thus, multi-parameter/multi-compound biomarker profiles may be identified that could mirror the combined change of multiple biological processes and biomolecular pathways upon any stress. The metabolic profiles indicated an increase in the effect of shading on the physiology of the plants with nutrient enrichment, which this effect becoming more prominently apparent in the combined profiles. Moreover, it was mainly the profile analysis that indicated the large difference in the physiology of the plants in the SH-NL5 conditions. This multivariate analysis result was a strong indicator that at this nutrient level, the plant has reached the limit of its tolerance for nutrient increase accommodation, to the extent that it experienced the change in the light availability due to shading in a severe way.

The combined profile analysis pointed to molecular functions that are expected to be activated during shading, connecting the concentration of chlorophylls with the drastic increase in the abundance of phytol at SH-NL5 conditions. In our study, as expected, individuals of shade treatments showed higher concentration of chlorophylls compared to the open treatment conditions (Valladares and Niinemets, 2008; Supplementary Figure S1). The plants responded to light availability by changes in their photosynthetic apparatus, including changes in the composition of the photosynthetic pigments (Björkman, 1981). In low light conditions, plants tend to maximize light absorption by increasing the production of chlorophyll. Phytol is a diterpene alcohol, which is the prenyl side chain of chlorophyll and is derived from geranylgeraniol (Gutbrod et al., 2019). Recent results demonstrated that the conversion of geranylgeraniol to phytol is linked to chlorophyll synthesis (Gutbrod et al., 2019). Ischebeck et al. (2006) showed that free phytol produced by chlorophyll hydrolysis could be re-incorporated into chlorophyll, or employed for the synthesis of tocopherol and fatty acid phytyl esters. Despite the significant increase in Chl-*a* concentrations in shade leaves, the Chl *a*:*b* ratio was similar between open and shade leaves. These results agree with previous studies that demonstrate that some species do not show significant changes in Chl *a*:*b* under limited light conditions (Zivcak et al., 2014; Ferroni et al., 2016).

### SLA, *φ* and Leaf Lactate Abundance Emerged as a Shading Acclimation Biomarker Measurement Set

Multivariate significance analysis in search of the factors characterizing the differences in the combined profiles of the plants due to shading revealed the cluster of SLA, φ and leaf lactate abundance as the most discriminatory, independently of the nutrient level conditions (Figures 4, 6; Supplementary Figure S5). Plants develop a higher SLA under low light conditions (Rozendaal et al., 2006; Valladares and Niinemets, 2008; Poorter et al., 2019) as a plastic response enabling a high photosynthetic performance under shading (Valladares and Niinemets, 2008; van Kleunen et al., 2011; Feng and van Kleunen, 2014). The increase in the photosynthetic efficiency of the plants was observed through the quantum yield (φ; Valladares and Niinemets, 2008). However, the role of leaf lactate abundance as a shading acclimation biomarker has not been indicated before, being a new contribution of the present study. Lactate is an oxidative stress related molecule (Rivoal et al., 1991; Dolferus et al., 2008; Nakamura and Noguchi, 2020). While its root abundance has been associated with hypoxic conditions (Dolferus et al., 2008; Maurino and Engqvist, 2015), the role of leaf lactate is not yet fully understood (Maurino and Engqvist, 2015), having been associated mainly with the plant protection from pathogens. In an older study in lettuce (Betsche, 1981), the production of leaf lactate was associated with the reducing equivalent and pH homeostasis, supporting thus the lactate-oxidative stress relationship. Our study provided strong evidence for its connection with light limitation. Furthermore, it revealed SLA, φ, and leaf lactate abundance, as quantitative markers of the level of light limitation under increased nutrient conditions (Figure 5). Interestingly, we observed an unknown metabolite, potentially sugar (Un_240), exhibiting the exact opposite profile, its abundance decreasing with shading with the decrease becoming larger from NL1 to NL5. These findings are important, as they open the discussion for the use of the combined SLA, φ, and leaf lactate abundance measurement set as a sensitive tool in large-scale environmental studies, for the evaluation of the level of strain inflicted on amphibious plants due to nutrient overloading and low light availability in real-life conditions.

### Combined Metabolic With Ecophysiological Profiling Is a Sensitive Tool for Stream Ecological Research

Integrated morpho-physiological and omic studies, using multivariate computational analyses for data mining and measurement interpretation, are not a common approach in the study of plant responses to environmental factors, not only for amphibious plants but also in general. Their value for sensitive and accurate monitoring of plant physiology compared to more classical approaches is gaining support and is expected to evolve as the established way of performing ecophysiological studies (Sardans et al., 2011; Arbona et al., 2013; Peters et al., 2018). In the present work, the metabolic profiles of the shade mesocosms were clearly distinguished from those in the open treatments independently of the nutrient level. This discriminatory power was higher when high-throughput metabolic data were combined with ecophysiological profiles. In addition, the multivariate analysis of the combined profiles supported the importance of factors, which was not directly apparent from the morpho-physiological measurement set alone.

Our results underline the importance of integrated multivariate analyses of plant physiological performance, morphology, and metabolic activity to provide mechanistic evidence of how multiple environmental challenges affect the adaptive capacity and shape the physiology of the plants. One of the current challenges in the field of stream ecology is to identify the effects of global environmental changes on biodiversity, ecosystem functioning, and services, which are central to environmental policy and effective management strategies. Ecological theory suggests that dynamic systems respond smoothly to environmental factors; however, profound changes often occur after a critical threshold, a turning point, at which the system shifts suddenly from one state to another over a range of conditions (Scheffer et al., 2001; Scheffer and Carpenter, 2003). Thus, the application of metabolomics in ecology (eco-metabolomics) and high-throughput profiling analysis, in general, promises to reveal the missing links between plant phenotypic responses and molecular mechanisms (Peters et al., 2018). Shifts in multi-factor/multi-compound profiles can serve as an “early warning signal”, facilitating the prediction of sudden deterioration before the critical environmental transition occurs. The challenge of the ecological research is to adapt these analytical protocols and extrapolate the acquired results from mesocosm studies into open field experiments and models.

To conclude, our results through integrated multivariate analysis of eco-physiological and metabolomic measurements underline the usefulness of this methodological framework in stream ecology research, revealing plant adaptation mechanisms to environmental challenges. Metabolic profile shifts could be used as “early warning” biomarkers of challenging conditions, enabling the timely and effective application of stream management and restoration methods.

## Data Availability Statement

The original contributions presented in the study are included in the article/Supplementary Material, further inquiries can be directed to the corresponding authors.

## Author Contributions

PM and TR conceived the ideas and designed the experiment and the eco-physiological analyses. PM, GT, and MK conceived and designed the metabolomic analysis approach, performed the interpretation of the dataset and wrote the manuscript. MK coordinated the metabolomic analysis and secured funding for the metabolomic and bioinformatic analyses. PM, FE, BS, and CB performed the acquisition of the data for plant physiology. GT performed the metabolomic data acquisition. PM coordinated the analyses of the plant physiology data and secured funding for the experiment and eco-physiological study through Marie-Curie fellowship grant. MK and GT carried out the multivariate statistical analysis of the metabolomic and combined profiles. TR, BS, and FE contributed critically to manuscript reviewing and editing. PM, GT, and MK took all authors’ constructive comments into account and finalized the manuscript. All authors contributed to the article and approved the submitted version.

### Conflict of Interest

The authors declare that the research was conducted in the absence of any commercial or financial relationships that could be construed as a potential conflict of interest.
