# Supplementary material for: Probing the Response of the Amphibious Plant Butomus umbellatus to Nutrient Enrichment and Shading by Integrating Eco-Physiological With Metabolomic Analyses
Source: Front Plant Sci. 2020 Dec 16;11:581787. doi: 10.3389/fpls.2020.581787 (PMC7772459; doi:10.3389/fpls.2020.581787)
Supplement: Supplementary file 1 [file Data_Sheet_1.pdf]

Table of Contents

Table S1: Morpho-physiological parameters and method(s) of measurement/estimate 2

Table S2: The normalized metabolomic dataset 3

Table S3: Mean values and standard deviation (± SD) of all morpho-physiological parameters 4

Figure S1: Scatter (XY) plots with standard deviation (± SD) of morpho-physiological parameters. 6

Figure S2: Number of hours per different Photosynthetic Active Radiation (PAR) range for open and shade treatments.. 7

Figure S3: Light response-curves for open and shade treatments 8

Figure S4: Hierarchical clustering analysis (HCL) of the standardized leaf metabolic profiles (Euclidean distance metric). 9

Figure S5: The set of metabolites identified as positively (POS) and negatively (NEG) significant in the shade versus the open treatments, based on SAM. 10

Figure S6: The abundance profiles of the 6 significant metabolites (5 positive, 1 negative) shown in Figure S5. 11

Figure S7: PCA graph of the standardized combined profiles. 12

Table S1: Morpho-physiological parameters and method(s) of measurement/estimate

Morpho-physiological parameter	Abbreviation (units)	Method of measurement
Above ground biomass dry mass	Above Ground Biomass (DW, g)	Freeze-dried dry weight of the above ground part of the individuals, after harvesting
Below ground biomass dry mass	Below Ground Biomass (DW, g)	Freeze-dried dry weight of the below ground part of the individuals, after harvesting
Plant height	PH (cm)	Final plant above ground height, after harvesting
Number of leaves	Number of leaves	Number of leaves after harvesting
Relative Growth Rate	RGR (g DW day^{-1})	Based on the mean of natural logarithm transformed dry weights according to Hoffmann and Poorter (2002). DW_{start} was estimated from the measured fresh weight (FW_{start}) at the beginning of the experiment, based on the proportionality coefficient that was calculated from the FW and DW measurements (below and above ground; 60 °C) of 10 morphologically identical replicates not used in the mesocosms (15 cm height). RGR = [ln (DW_{end}) – (lnDW_{start})] / (t_{days})
Chlorophyll *a* concentration and *b*	Chl-*a;* Chl-*b* (mg DWg^{-1})	Photosynthetic pigments concentrations were determined spectrophotometrically after extraction overnight in the darkness in 96% ethanol according to Lichtenthaler (1987).
Ratio Chlorophyll *a* concentration: *b*	Chl *a:b*	
Carotenoids concentration	Carotenoids (mg DWg^{-1})	
Specific leaf area	SLA (cm^2 g^{-1})	Ratio of leaf area used to leaf dry mass; measurements refer to the leaf used for photosynthesis measurements
%N per leaf DW	%LeafN	Tissue content (percentage per dry weight) of carbon and nitrogen were determined for all leaves used for photosynthesis measurements, above ground organs (shoot) and below ground organs (root) for each individual which means triplicate samples of each treatment by catalytic combustion at 900 ◦C on a C/N analyzer (CE Instruments).
%C per leaf DW	%LeafC	
%N per Root DW	%RootN	
%C per Root DW	%RootC	
%N per Shoot DW	%ShootN	
%C per Shoot DW	%ShootC	
Ratio C:N in root	C:N_root	Ratio between %C content per dry weight and %N root (below ground) content per dry weight
Ratio C:N in leaf	C:N_leaf	Ratio between %C content per dry weight and %N leaf content per dry weight
Ratio C:N in Shoot	C:N_shoot	Ratio between %C content per dry weight and %N shoot (above ground) content per dry weight
N content in the leaf	FoliarN (mgN)	Concentration of N in mgN in the leaf used for photosynthesis
Photosynthetic N use efficiency	PNUE; (μmolCO_2/gN/s)	Maximum Photosynthesis rate (P_{max}) per unit leaf area divided by leaf N concentration. (Photosynthetic rate X SLA) / (Foliar N concentration) (Hidaka and Kitayama, 2009)
Maximum Photosynthesis rate	P_{max}; [μmol (CO_2) m^{-2}s^{-1}]	From light response curve fitted using rectangular hyperbola Michaelis-Menten model (Baly, 1935)
Light compensation point	Ic; [μmol (photons) m^{-2} s^{-1}]	From light response curve fitted using rectangular hyperbola Michaelis-Menten model (Baly, 1935)
Quantum yield	φ; [mmol (CO_2) mmol^{-1} (photons)]	From light response curve fitted using rectangular hyperbola Michaelis-Menten model (Baly, 1935)
Dark respiration rate	Rd; [μmol (CO_2) m^{-2} s^{-1}]	From light response curve fitted using rectangular hyperbola Michaelis-Menten model (Baly, 1935)
Light saturation point	Ik; [μmol (photons) m^{-2}s^{-1}]	From light response curve fitted using rectangular hyperbola Michaelis-Menten model (Baly, 1935)
Maximum gross photosynthesis rate	G_{max}; [μmol (CO_2) m^{-2}s^{-1}]	From light response curve fitted using rectangular hyperbola Michaelis-Menten model (Baly, 1935)

Table S2: The normalized metabolomic dataset

	Peak Name	Metabolite Name	RT	Quan Ion	Chem. Cat.*	fold change OPEN_NL4 over OPEN_NL1	fold change OPEN_NL5 over OPEN_NL1	fold change SH_NL1 over OPEN_NL1	fold change SH_NL4 over OPEN_NL4	fold change SH_NL5 over OPEN_NL5	OPEN_NL1_R1	OPEN_NL1_R2	OPEN_NL1_R3	SH_NL1_R1	SH_NL1_R2	SH_NL1_R3	OPEN_NL4_R1	OPEN_NL4_R2	OPEN_NL4_R3	SH_NL4_R1	SH_NL4_R2	SH_NL4_R3	OPEN_NL5_R1	OPEN_NL5_R2	OPEN_NL5_R3	SH_NL5_R1	SH_NL5_R2	SH_NL5_R3
1	3-methylbenzoate 1TMS	3-methylbenzoate	18.302	193	1	0.97	1.59	1.93	1.17	0.68	0.029	0.057	0.028	0.033	0.047	0.032	0.030	0.034	0.031	0.038	0.028	0.031	0.033	0.038	0.038	0.037	0.035	0.041
2	alpha-linolenic acid 1 TMS	alpha-linolenic acid	34.858	79	1	1.19	1.64	0.94	0.91	0.90	0.063	0.059	0.074	0.068	0.103	0.093	0.054	0.077	0.050	0.026	0.052	0.034	0.046	0.057	0.067	0.077	0.064	0.080
3	caffeic acid 3TMS	caffeic acid	33.474	219.1	1	0.45	1.15	0.09	1.69	1.22	1.601	0.147	0.728	1.228	1.839	2.243	0.978	0.840	1.335	0.820	0.339	0.826	0.742	1.003	0.930	0.621	0.601	0.784
4	citrate 4TMS	citrate	26.596	273.3	1	1.25	0.84	0.08	0.23	0.28	4.136	0.332	5.172	1.205	3.465	0.958	0.781	0.619	1.074	0.774	1.116	1.632	0.586	1.591	0.628	1.220	0.918	1.521
5	ethanolamine 3TMS	ethanolamine 3TMS	11.166	174.2	3	0.44	0.26	0.90	1.73	0.13	0.048	0.043	0.021	0.036	0.012	0.002	0.065	0.061	0.009	0.073	0.036	0.025	0.061	0.023	0.019	0.182	0.110	0.146
6	fructose MeOx 1	fructose MeOx 1	25.829	307.1	2	2.77	1.56	0.70	0.38	1.48	0.684	0.480	1.894	0.726	1.064	1.570	4.482	3.775	1.552	1.728	3.550	2.860	3.385	3.970	3.496	1.993	2.939	3.337
7	fumarate 2TMS	fumarate	15.867	245	1	0.47	0.54	0.19	1.52	0.73	0.214	0.041	0.100	0.152	0.115	0.084	0.165	0.206	0.309	0.201	0.111	0.246	0.169	0.299	0.199	0.348	0.255	0.260
8	gluconate 6 TMS	gluconate	27.886	292.2	1	0.97	0.78	0.52	0.41	0.68	0.065	0.034	0.063	0.026	0.050	0.034	0.062	0.057	0.073	0.040	0.062	0.059	0.073	0.087	0.066	0.073	0.059	0.057
9	glucose total	glucose total			2	2.53	1.11	0.75	0.26	1.33	4.501	3.386	11.380	3.014	5.015	6.670	15.103	20.338	32.441	11.462	17.516	19.342	19.466	21.985	21.841	13.437	17.364	21.384
10	glutamate_total	glutamate_total			3	1.25	0.55	0.44	0.18	2.10	0.320	0.141	0.399	0.070	0.176	0.369	1.036	0.713	0.301	0.323	0.871	0.617	0.846	0.679	0.572	1.544	1.339	0.589
11	glycerate 3TMS	glycerate	14.818	292	1	1.01	1.04	0.43	0.84	1.02	0.158	0.068	0.159	0.134	0.163	0.166	0.193	0.397	0.485	0.328	0.248	0.281	0.374	0.490	0.374	0.682	0.680	0.426
12	glycerol 3TMS	glycerol	12.002	204.9	1	1.04	2.31	1.74	0.73	0.66	0.017	0.030	0.018	0.013	0.040	0.026	0.023	0.031	0.029	0.040	0.057	0.032	0.034	0.037	0.035	0.049	0.040	0.030
13	lactate 2 TMS	lactate	6.989	117.2	1	0.52	2.37	0.66	2.87	0.90	0.063	0.042	0.033	0.095	0.151	0.136	0.044	0.049	0.035	0.061	0.141	0.114	0.028	0.040	0.029	0.041	0.070	0.068
14	9,12-(Z,Z)-Octadecadienoic acid (1TMS)	linoleic acid	34.372	337.3	1	1.23	1.22	1.16	0.61	0.89	0.006	0.006	0.007	0.004	0.007	0.006	0.006	0.010	0.004	0.001	0.010	0.008	0.004	0.006	0.011	0.006	0.010	0.016
15	malate 3TMS	malate	19.495	265.2	1	0.82	0.85	0.17	1.09	0.42	0.289	0.049	0.237	0.258	0.246	0.103	0.449	0.650	1.175	0.817	0.563	0.775	0.544	1.194	0.558	1.555	1.166	1.276
16	myo-inositol 6TMS	myo-inositol	28.918	305.3	1	1.40	1.75	0.65	0.49	0.59	5.680	3.689	7.951	3.859	9.947	5.893	10.007	10.933	10.533	13.397	11.727	13.267	13.709	10.970	13.502	8.061	12.993	12.949
17	octadecanoic acid 1TMS	octadecanoic acid	34.08	117.1	1	1.85	2.86	3.19	0.62	0.53	0.056	0.178	0.103	0.064	0.159	0.085	0.067	0.145	0.066	0.063	0.078	0.077	0.057	0.108	0.107	0.074	0.071	0.150
18	P0802 (organic acid)	P0802 (organic acid)	7.851	131.2	1	1.82	3.29	2.94	0.78	0.55	0.013	0.039	0.024	0.019	0.044	0.024	0.024	0.018	0.027	0.033	0.018	0.015	0.029	0.032	0.025	0.028	0.030	0.023
19	P1188	P1188	11.729	131	1	1.05	1.73	1.63	1.05	0.69	0.017	0.027	0.018	0.019	0.029	0.020	0.018	0.018	0.019	0.026	0.018	0.015	0.021	0.023	0.022	0.023	0.023	0.021
20	phosphate 4TMS	phosphate	14.565	299.2	1	1.00	1.13	1.31	1.84	1.05	0.248	0.324	0.248	0.455	0.279	0.294	0.459	0.429	0.320	0.614	0.480	0.347	0.565	0.481	0.285	1.779	1.126	0.687
21	phytol 1 TMS (3-7-11-15)	phytol	32.223	143	1	1.29	2.11	0.84	0.63	0.66	0.017	0.014	0.022	0.014	0.036	0.024	0.027	0.040	0.017	0.027	0.047	0.028	0.033	0.029	0.030	0.076	0.041	0.050
22	putrescine 4 TMS	putrescine	22.157	174.2	3	0.69	0.36	0.49	0.29	0.78	0.074	0.037	0.051	0.015	0.026	0.021	0.069	0.057	0.075	0.073	0.125	0.062	0.105	0.087	0.066	0.080	0.069	0.062
23	serine 2 TMS	serine 2 TMS	14.187	116.2	3	0.48	0.88	0.26	0.51	0.35	0.026	0.007	0.013	0.006	0.023	0.008	0.012	0.014	0.004	0.014	0.038	0.024	0.006	0.005	0.002	0.055	0.053	0.011
24	succinate 2TMS	succinate	16.04	246.8	1	0.80	0.73	0.24	0.79	0.78	0.046	0.011	0.037	0.029	0.033	0.026	0.082	0.088	0.146	0.067	0.057	0.073	0.055	0.168	0.079	0.159	0.051	0.092
25	sucrose 8 TMS	sucrose	38.215	437.1	1	1.37	1.39	0.84	0.51	0.70	10.434	8.740	14.318	7.369	14.455	10.131	16.727	15.330	16.787	19.251	15.382	19.895	20.458	18.048	18.937	18.334	18.362	19.688
26	threonate 4TMS	threonate	20.279	292.2	1	1.80	0.65	1.30	0.20	1.33	0.160	0.207	0.287	0.058	0.103	0.137	0.255	0.260	0.184	0.126	0.352	0.162	0.404	0.237	0.257	0.268	0.320	0.265
27	Tom30.24 (sugar)	Tom30.24 (sugar)	29.634	319.1	1	2.58	2.08	0.34	0.38	0.80	0.200	0.069	0.517	0.199	0.417	0.335	0.674	0.716	0.271	0.433	0.489	0.269	0.632	0.601	0.664	0.276	0.443	0.498
28	Un_0028 (P2746)	Un_0028 (P2746)	27.234	123.1	1	1.37	1.43	0.99	0.68	0.81	0.009	0.008	0.012	0.008	0.012	0.010	0.009	0.014	0.008	0.012	0.012	0.011	0.015	0.009	0.012	0.015	0.018	0.017
29	Un_0050 (P2509,unknown_no111) (sugar)	Un_0050 (P2509,unknown_no111) (sugar)	24.931	217.1	1	1.44	2.02	0.98	0.54	0.79	0.203	0.198	0.293	0.157	0.411	0.323	0.212	0.150	0.189	0.075	0.134	0.141	0.113	0.171	0.271	0.084	0.105	0.180
30	Un_0096 (P1552, (amino) acid)	Un_0096 (P1552, (amino) acid)	15.333	174.2	3	0.67	1.37	1.10	0.99	0.25	0.199	0.220	0.133	0.131	0.273	0.069	0.123	0.117	0.106	0.142	0.137	0.135	0.117	0.191	0.126	0.121	0.132	0.085
31	Un_0134 (P2957,Tom29.96, sugar pyranos	Un_0134 (P2957,Tom29.96, sugar pyranose)	29.385	204.1	1	4.35	1.03	0.70	0.15	1.46	0.098	0.069	0.426	0.062	0.101	0.148	0.592	0.742	0.249	0.357	0.752	0.486	0.538	0.695	0.547	0.327	0.673	0.683
32	Un_0228 (P1754)	Un_0228 (P1754)	17.047	228.2	1	0.90	1.32	1.18	0.95	0.67	0.006	0.008	0.006	0.005	0.008	0.006	0.005	0.005	0.005	0.007	0.006	0.006	0.006	0.006	0.006	0.005	0.006	0.006
33	Un_0233 (P3353)	Un_0233 (P3353)	33.346	331	1	1.26	1.25	0.52	0.58	0.92	0.014	0.007	0.018	0.010	0.018	0.016	0.028	0.020	0.016	0.027	0.023	0.023	0.026	0.037	0.019	0.017	0.021	0.032
34	Un_0236 (Tom26.5)	Un_0236 (Tom26.5)	26.229	123.1	1	1.54	1.82	1.06	0.69	0.63	0.023	0.025	0.036	0.025	0.042	0.027	0.025	0.047	0.026	0.036	0.038	0.038	0.047	0.039	0.042	0.045	0.055	0.054
35	Un_0242 (Un_0230 MeOx 2) (Tom19.13)	Un_0242 (Un_0230 MeOx 2,Tom19.13)	18.876	171.9	2	3.68	1.46	0.71	0.53	0.89	0.003	0.002	0.012	0.006	0.005	0.004	0.005	0.017	0.031	0.012	0.003	0.020	0.013	0.012	0.029	0.006	0.008	0.019
36	Un_0243 (Tom30.19)	Un_0243 (Tom30.19)	29.633	376.1+262.3	1	2.32	1.87	0.55	0.50	1.09	0.031	0.017	0.072	0.036	0.058	0.063	0.094	0.101	0.039	0.058	0.092	0.078	0.091	0.085	0.090	0.039	0.064	0.071
37	Un_0249 (putative 2-ketogluconic acid)	Un_0249 (putative 2-ketogluconic acid)	23.168	333.2	2	0.90	1.37	0.19	0.82	0.62	0.078	0.015	0.070	0.057	0.107	0.067	0.077	0.099	0.132	0.089	0.066	0.120	0.106	0.132	0.115	0.110	0.109	0.112
38	Un_240 (Tom31.07) sugar	Un_240 (Tom31.07) sugar	30.513	319.1	1	2.29	1.26	0.71	0.35	1.21	0.096	0.069	0.221	0.076	0.122	0.148	0.178	0.212	0.101	0.082	0.156	0.132	0.189	0.157	0.215	0.057	0.086	0.110
39	xylitol 5 TMS	xylitol	22.286	103.2+217.2	1	2.28	0.85	1.70	0.50	0.67	0.067	0.114	0.152	0.075	0.057	0.038	0.280	0.230	0.146	0.424	0.206	0.035	0.200	0.222	0.225	0.133	0.119	0.117
	**PEAK AREA OF INTERNAL STANDARD (in counts)**																											
	ribitol 5TMS	ribitol	21.977	217.1	1						407882	308105	440175	428334	269926	417642	485215	193064	449058	316481	418607	340942	319231	414313	367887	504681	412798	386844

Notes:

For the unknown metabolites the number in the MESBL database is provided and in parenthesis previously assigned by our group annotations for the same peak that have been included in published reports and publications.

* **Chemical Category** refers to the categorization with respect to the number and type of derivatives for a metabolite as defined in Kanani and Klapa, 2007.

Table S3: Mean values and standard deviation (± SD) of all morpho-physiological parameters

	TREATMENTS									
**Traits**	**SH-NL1**	**SH-NL2**	**SH-NL3**	**SH-NL4**	**SH-NL5**	**OPEN-NL1**	**OPEN-NL2**	**OPEN-NL3**	**OPEN-NL4**	**OPEN-NL5**
**Above Ground Biomass** (DW, g)	0.34±0.078	0.97±0.42	1.04±0.66	3.29±0.47	4.00±1.37	0.26±0.06	0.96±0.45	1.43±0.84	2.72±1.67	3.17±1.67
**Below Ground Biomass** (DW, g)	0.84±0.52	2.38±1.02	2.63±1.30	8.61±1.34	8.57±4.09	1.08±0.26	3.01±1.52	3.62±1.99	5.61±2.60	9.11±4.24
**Plant height** (PH, cm)	31.89±0.69	33.67±1.53	47.22±10.18	53.56±3.36	56.22±9.79	24.56±5.01	35.00±7.17	32.33±7.37	43.56±3.59	47.44±6.54
**Number of leaves**	4.67±0.58	10.00±1.73	9.33±2.08	17.00±6.00	20.67±3.51	5.67±1.53	10.67±1.15	15.00±1.00	20.33±11.15	20.33±6.81
**RGR** [DW(g/days)]	0.02±0.01	0.03±0.01	0.03±0.01	0.05±0.00	0.05±0.01	0.02±0.00	0.03±0.01	0.04±0.01	0.04±0.01	0.05±0.01
**Chl-*a*** (mg DWg^{-1})	4.46±0.73	8.06±0.63	7.92±0.32	8.12±1.12	9.52±1.71	3.98±0.98	4.26±1.40	6.47±0.55	6.12±0.71	6.95±0.96
**Chl-*b*** (mg DWg^{-1})	1.58±0.21	2.74±0.22	2.68±0.12	2.68±0.32	3.13±0.64	1.50±0.27	1.47±0.45	2.12±0.17	2.08±0.25	2.25±0.24
**Chl-*a*+*b*** (mg DWg^{-1})	6.03±0.93	10.80±0.85	10.60±0.43	10.80±1.44	12.65±2.35	5.48±1.25	5.73±1.85	8.58±0.72	8.20±0.95	9.20±1.18
**Chl *a:b***	2.82±0.087	2.94±0.018	2.96±0.029	3.03±0.09	3.05±0.09	2.64±0.17	2.89±0.07	3.05±0.04	2.94±0.11	3.08±0.17
**Carotenoids** (mg DWg^{-1})	0.71±0.24	1.26±0.43	1.31±0.57	1.86±0.38	2.24±0.39	0.50±0.54	0.85±0.13	1.16±0.45	1.29±0.07	1.66±0.21
**Specific Leaf Area, SLA** (cm^2 g^{-1})	187.32±58.88	191.74±14.24	197.13±73.60	185.72±21.06	142.52±38.63	171.63±28.51	183.42±22.11	163.42±13.93	125.63±11.14	132.04±24.76
**%N per leaf DW** (%LeafN)	2.19±0.17	3.08±0.27	3.05±0.15	3.16±0.61	3.54±0.65	2.59±0.31	2.67±0.35	3.07±0.27	2.88±0.15	3.08±0.59
**%C per leaf DW** (%LeafC)	38.02±1.81	41.22±1.16	40.50±1.17	40.64±1.48	40.75±2.31	38.50±1.88	39.78±0.45	40.78±2.68	39.95±1.16	42.09±1.50
**%N per Root DW** (%RootN)	0.77±0.26	1.36±0.15	1.47±0.13	1.30±0.025	1.95±0.36	0.78±0.051	1.15±0.30	1.23±0.25	1.09±0.14	1.48±0.44
**%C per Root DW** (%RootC)	38.36±2.68	39.41±1.87	39.55±0.53	35.39±1.67	38.26±2.84	36.55±1.81	37.10±4.01	37.35±2.94	37.87±1.96	37.87±3.45
**%N per Shoot DW** (%ShootN)	0.77±0.25	1.56±0.27	1.62±0.25	1.78±0.22	2.01±0.41	0.84±0.12	1.35±0.20	1.51±0.18	1.34±0.21	1.75±0.34
**%C per Shoot DW** (%ShootC)	31.95±4.72	36.25±1.22	36.40±0.77	36.42±0.33	36.73±0.46	31.93±3.89	37.22±0.29	37.34±0.73	35.52±2.44	36.90±0.75
**C:N root**	53.00±13.90	29.37±4.34	27.11±2.43	27.31±1.81	20.05±3.73	46.88±5.09	33.09±5.37	31.06±5.71	35.02±3.36	26.49±5.09
**C:N_leaf**	17.41±1.74	13.41±0.80	13.30±0.32	13.12±1.94	11.67±1.40	14.97±1.03	15.10±2.30	13.35±1.46	13.87±0.56	14.00±2.53
**C:N_shoot**	42.86±7.30	23.66±3.74	22.80±3.26	20.67±2.65	18.81±3.95	38.49±5.22	27.98±4.15	24.94±3.41	26.76±3.58	21.60±4.09
**FoliarN** (mgN)	0.10±0.01	0.11±0.01	0.13±0.01	0.16±0.02	0.21±0.02	0.10±0.03	0.13±0.03	0.12±0.02	0.16±0.14	0.20±0.04
**PNUE** (μmolCO$_2$/gN/s)	4035.59±700.15	5258.29±120.51	4394.53±1474.91	3532.65±295.72	2285.03±480.86	5292.05±2879.05	4095.06±769.32	5043.99±678.52	3112.33±439.51	2320.56±237.08
$\mathbf{P_{max}}$ [μmol(CO$_2$)m^{-2}s^{-1}]	10.84±4.36	17.29±4.86	18.26±8.18	22.46±2.43	22.01±4.26	12.39±7.02	16.51±3.99	20.09±6.73	23.58±3.56	22.27±8.28
$\mathbf{G_{max}}$ [μmol (CO$_2$) m^{-2}s^{-1}]	22.06±4.54	30.70±3.93	31.00±11.00	30.91±2.82	33.57±3.46	27.94±9.82	27.75±6.99	36.69±5.23	40.21±4.12	36.08±10.61
**Ic** [μmol (photons) m^{-2} s^{-1}]	54.82±43.31	50.45±30.46	49.91±35.58	10.52±4.57	22.25±9.26	86.87±39.76	30.82±13.92	62.32±60.09	31.48±16.47	36.37±21.67

**φ** [mmol (CO_2) $mmol^{-1}$ (photons)]	0.09±0.04	0.08±0.01	0.08±0.03	0.10±0.02	0.08±0.01	0.06±0.03	0.08±0.00	0.06±0.02	0.07±0.01	0.07±0.00
**Rd** [μmol(CO_2)m^{-2} s^{-1}]	3.37±1.31	3.23±1.43	3.00±0.69	1.00±0.26	1.53±0.45	4.00±0.10	2.30±0.95	2.90±2.97	1.97±0.83	2.17±1.07
**Ik** [μmol (photons) $m^{-2}s^{-1}$]	462.00±66.16	816.67±190.14	840.67±153.58	955.67±169.15	1071.33±224.63	619.67±140.23	711.33±153.68	950.33±84.13	1015.00±95.41	1150.67±332.54

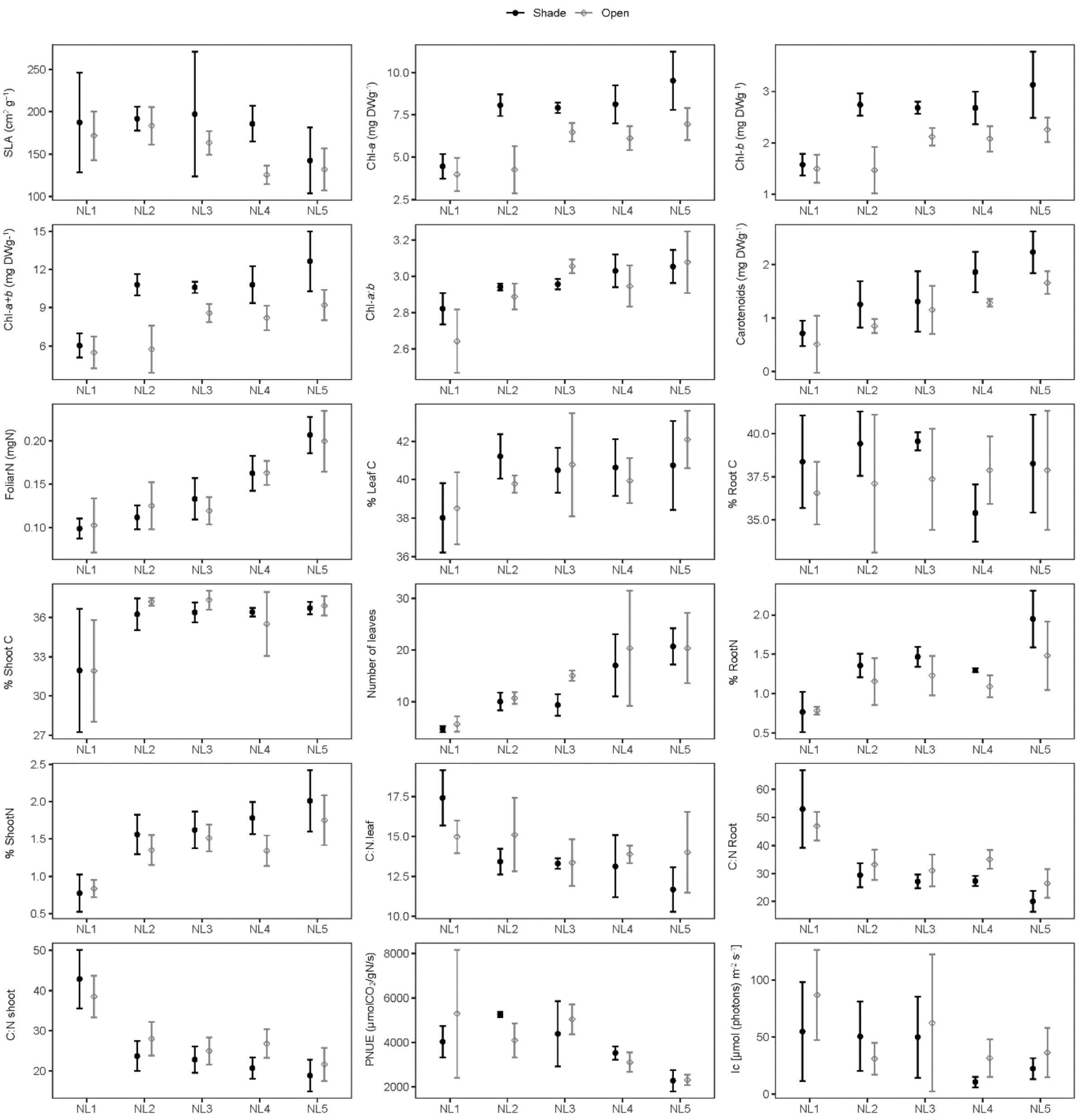

Figure S1: Scatter (XY) plots with standard deviation (± SD) of morpho-physiological parameters. Filled black circles: shaded treatments; gray open circles: open treatments. SD estimated over n=3 mesocosms per treatment.

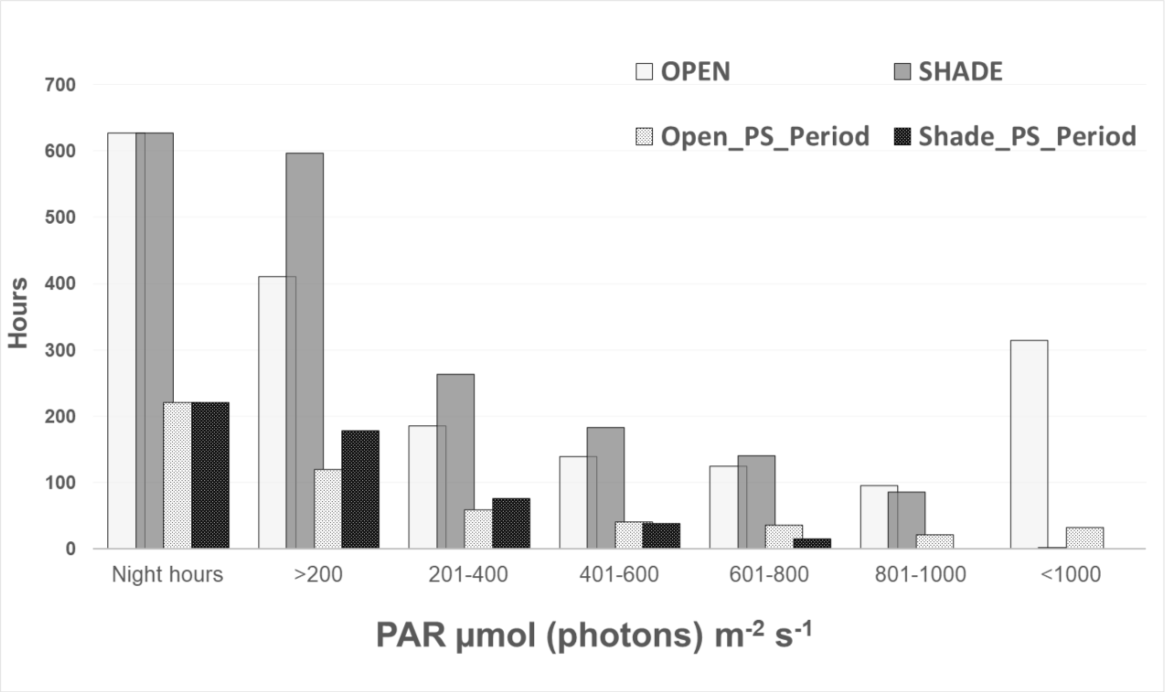

Figure S2: Number of hours per different Photosynthetic Active Radiation (PAR) range for open and shade treatments. Data were collected from the Påskehøjgård weather station and for conversion of KW m^{-2} to µmol photons m^{-2} s^{-1}, Langhans and Tibbitts (1997) was used for conversion.

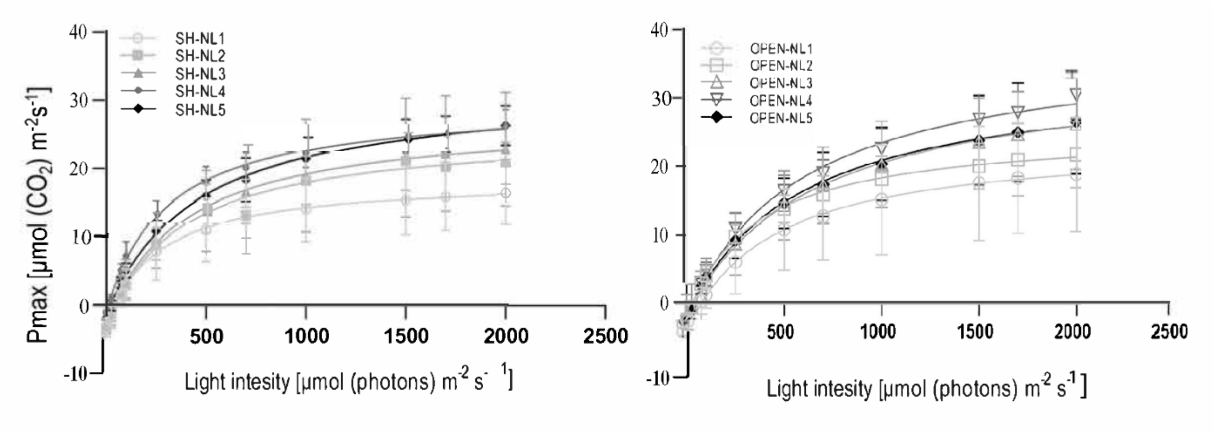

Figure S3: Light response-curves for open and shade treatments. Photosynthetic capacity was characterized from light response curves determined from measurements at eleven irradiances (2000, 1700, 1500, 1000, 700, 500, 250, 100, 75, 25 and 0 µmol $m^{-2}s^{-1}$). The light response curve of each leaf was fitted using rectangular hyperbola Michaelis-Menten based model of Baly (1935). Graphical representation of P/I curves was performed by GraphPad Prism 8.0.2 software (GraphPad Software, San Diego, CA, USA).

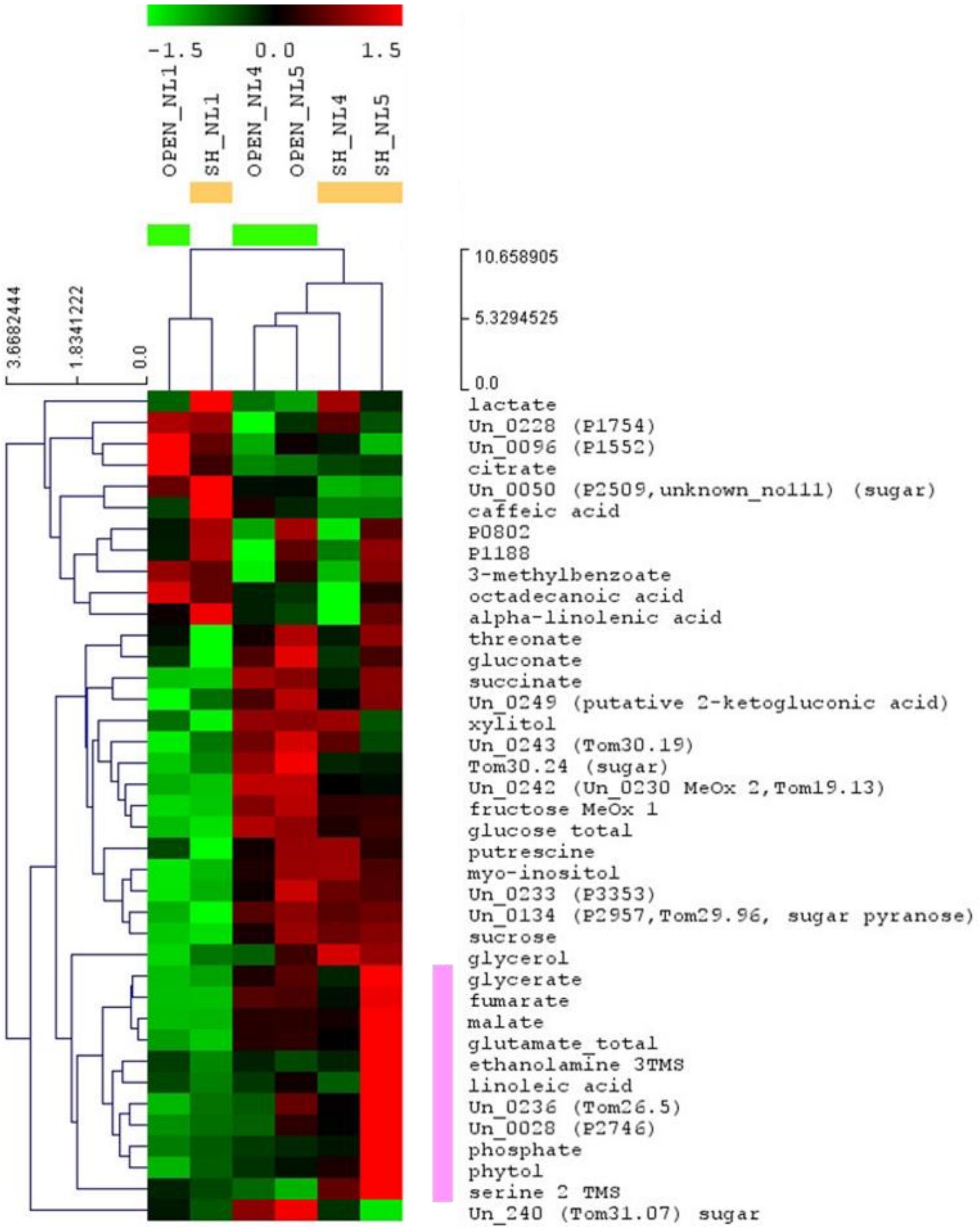

Figure S4: Hierarchical clustering analysis (HCL) of the standardized leaf metabolic profiles (Euclidean distance metric). Highlighted in pink is the cluster that comprises the metabolites that exhibiting a largely different abundance in SH-NL5.

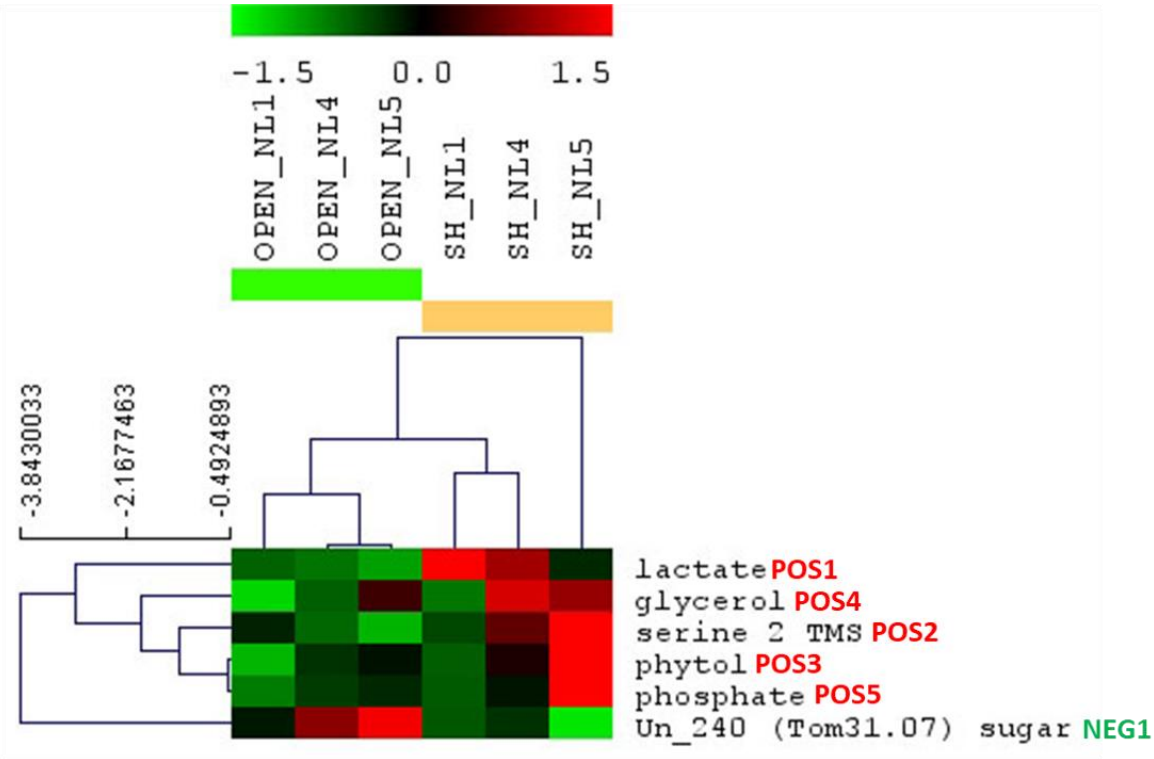

Figure S5: The set of metabolites identified as positively (POS) and negatively (NEG) significant in the shade versus the open treatments, based on SAM. The indication POS or NEG next to the name of each metabolite differentiates, respectively, the positively from the negatively significant in the shade compared to the open treatments; the number depicts the significance hierarchy of each metabolite in its respective group.

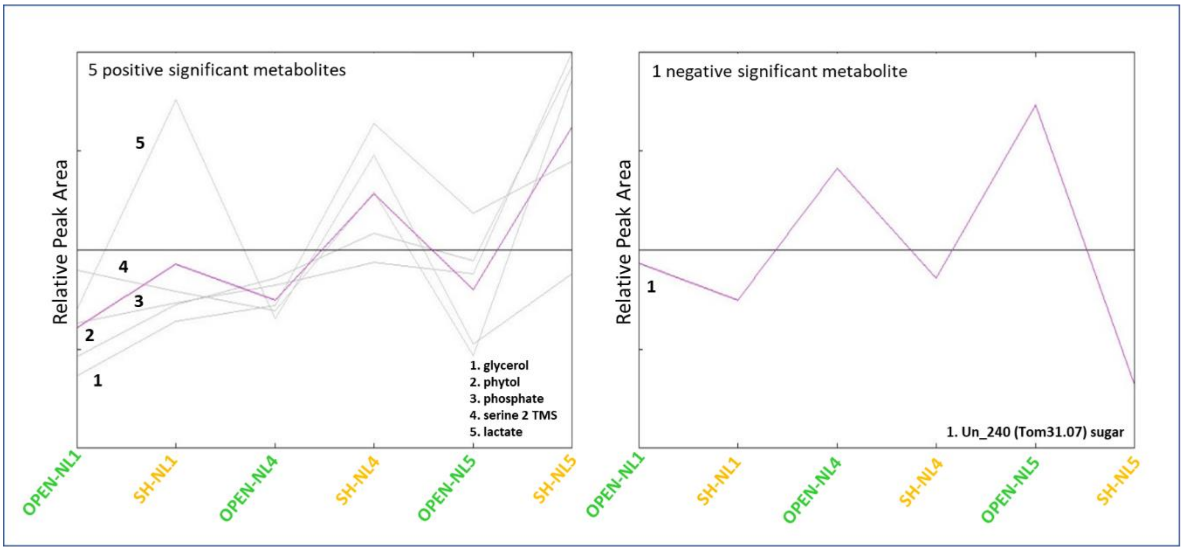

Figure S6: The abundance profiles of the 6 significant metabolites (5 positive, 1 negative) shown in Figure S5.

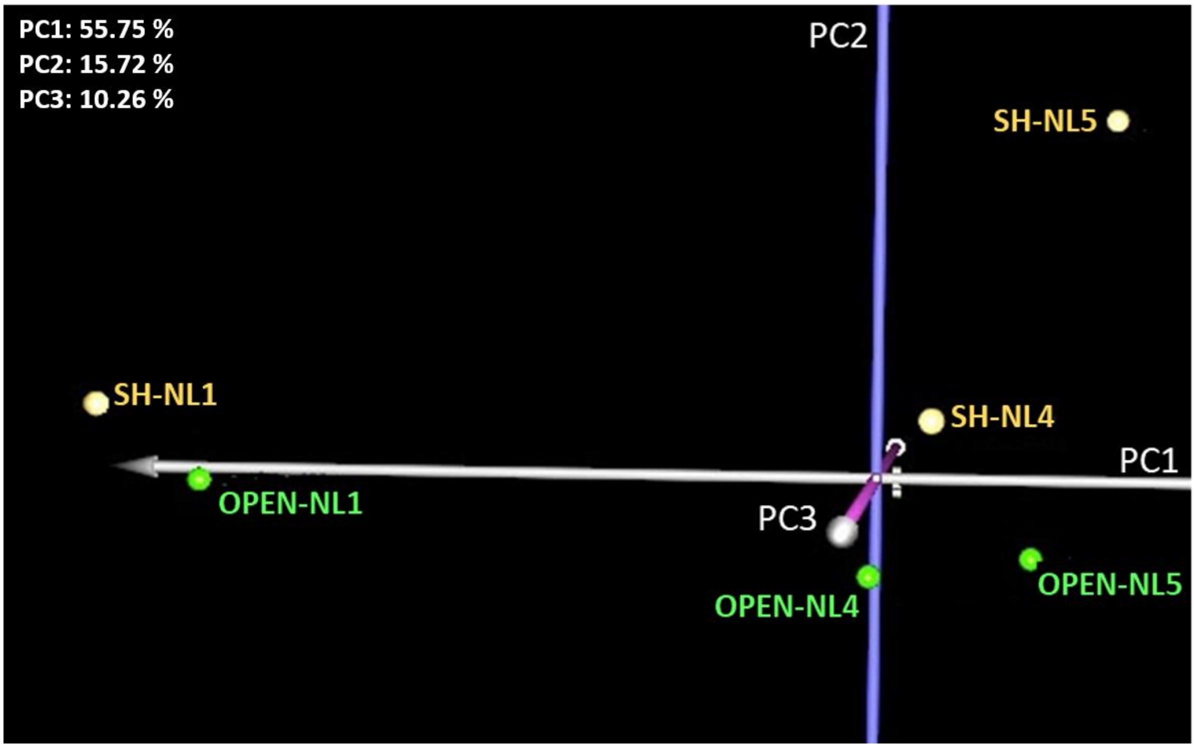

Figure S7: PCA graph of the standardized combined profiles.
